# Pan *msr* gene deleted strain of *Salmonella* Typhimurium suffers oxidative stress, depicts macromolecular damage and attenuated virulence

**DOI:** 10.1038/s41598-023-48734-w

**Published:** 2023-12-09

**Authors:** Raj Sahoo, Tapan Kumar Singh Chauhan, Lalhmangaihzuali Lalhmangaihzuali, Esha Sinha, Salauddin Qureshi, Manish Mahawar

**Affiliations:** 1 Division of Biochemistry, ICAR-IVRI, Izatnagar, 243122 India; 2 Division of Biological Standardization, ICAR-IVRI, Izatnagar, 243122 India

**Keywords:** Bacterial host response, Bacterial pathogenesis, Bacterial transformation, Immunoblotting, Thioredoxins, Oxidoreductases

## Abstract

*Salmonella* encounters but survives host inflammatory response. To defend host-generated oxidants, *Salmonella* encodes primary antioxidants and protein repair enzymes. Methionine (Met) residues are highly prone to oxidation and convert into methionine sulfoxide (Met-SO) which compromises protein functions and subsequently cellular survival. However, by reducing Met-SO to Met, methionine sulfoxide reductases (Msrs) enhance cellular survival under stress conditions. *Salmonella* encodes five Msrs which are specific for particular Met-SO (free/protein bound), and ‘*R*’/‘*S*’ types. Earlier studies assessed the effect of deletions of one or two *msrs* on the stress physiology of *S.* Typhimurium. We generated a pan *msr* gene deletion (Δ5*msr*) strain in *S.* Typhimurium. The Δ5*msr* mutant strain shows an initial lag in in vitro growth. However, the Δ5*msr* mutant strain depicts very high sensitivity (*p* < 0.0001) to hypochlorous acid (HOCl), chloramine T (ChT) and superoxide-generating oxidant paraquat. Further, the Δ5*msr* mutant strain shows high levels of malondialdehyde (MDA), protein carbonyls, and protein aggregation. On the other side, the Δ5*msr* mutant strain exhibits lower levels of free amines. Further, the Δ5*msr* mutant strain is highly susceptible to neutrophils and shows defective fitness in the spleen and liver of mice. The results of the current study suggest that the deletions of all *msrs* render *S.* Typhimurium highly prone to oxidative stress and attenuate its virulence.

## Introduction

Non-typhoidal *Salmonella* (NTS) are estimated to infect over 94 million people every year^1^. NTS infection manifests in a broad spectrum of diseases, including mild to modern gastroenteritis and fever. However, NTS can cause severe gastroenteritis, septicemia, and death in its fatal form^2,3^. The emergence of invasive^4,5^ and multidrug resistance^6^ strains impart additional challenges to the current treatment/ prophylaxis regimens.

Host defense against *Salmonella* infections involves a complex interplay of immune responses. Phagocytic cells, such as macrophages and neutrophils, play a very important role in controlling *Salmonella* infection. Initial inflammatory responses include the production of anti-microbial peptides (AMPs), reactive oxygen species (ROS), reactive nitrogen species (RNS), cytokines and chemokines. These immune mediators coordinate with each other not only to eliminate *Salmonella* but also to facilitate other immune cells in the elicitation of adaptive immune responses. Following activation of the phagocytes, NADPH oxidase (NOX) assembles on the phagosomal membrane and facilitates the transfer of electrons from NADPH to oxygen, leading to the generation of superoxide anions (O_2_^−^) anions^7^. The O_2_^−^ then metabolizes into other ROS such as hydrogen peroxide (H_2_O_2_), hydroxyl radicals (·OH) and hypochlorous acid (HOCl)^8^. The ROS can damage various bacterial macromolecules like proteins, lipids and DNA^9^. ROS-mediated damage to proteins includes covalent modifications to amino acids and protein unfolding. Unfolded proteins are prone to aggregation, and subsequently show compromised function^10^. Out of twenty constituent amino acids, the sulfur-containing amino acids, cysteine and methionine, are highly prone to oxidation^11^.

To counter host-generated oxidants, *Salmonella* harbors the T3SS system, antioxidants and repair enzymes^8^. T3SS effectors inhibit NADPH assembly and interfere with O_2_^−^ production^12^. The antioxidant enzymes, superoxide dismutases, catalases and peroxiredoxins catalytically degrade O_2_^−^ and H_2_O_2_^13,14^ The protein repair enzymes, Msrs reduce Met-SO back to Met^10,15^, whereas protein isoaspartyl methyl transferase repair of isoaspartate to aspartate^8^.

Msrs apparently serve two functions in the cell. First, by repairing Met-SO, Msrs modulate the activity of oxidized proteins. Second, Met residues (free or protein-bound) act as a sink to quench excess oxidants generated during host inflammatory response and get converted into Met-SO, which later get repaired by Msrs. Met-SO formation and their subsequent repair prevents ROS accumulation thereby ROS-mediated macromolecular damage in the cell and thus enhances cellular survival under oxidative stress.

*S.* Typhimurium encodes four cytoplasmic Msrs (MsrA, MsrB, MsrC and BisC) which are specific for particular types of Met-SO (free/ protein bound and "*R*"*/* "*S*" types). MsrA reduces the "*S*" form (free or protein-bound) of Met-SO. MsrB and MsrC repair protein-bound and free "*R*" Met-SO respectively. BisC repairs biotin sulfoxides and free "*S*" Met-SO. The contribution of MsrA, MsrC, and BisC in stress survival and virulence of *S.* Typhimurium is well documented^10,16–19^. However, MsrB does not play a vital role in this bacterium^16^.

Periplasmic proteins are involved in various physiological functions. However, due to location, they are highly prone to oxidation. *S.* Typhimurium expresses one dedicated Msr (MsrP) to repair proteins in this compartment^20^. A recent study demonstrated the role of MsrP in mitigating N-chlorotaurine induced envelope stress^15^. Further, the same study established the role of the CpxRA two-component system in sensing N-chlorotaurine and regulating MsrP expression. Subsequently, we observed a moderate role of MsrP against ChT-induced oxidative stress, neutrophil-mediated killing, and virulence in mice^21^.

As each Msr contributes (to some extent) to oxidative stress survival of *S.* Typhimurium, we hypothesized that the pan *msr* gene deletion strain might show very high sensitivity to oxidants, depict macromolecular damage and attenuated virulence.

## Results

### Confirmation of Δ5*msr* mutant strain

**Figure 1:**
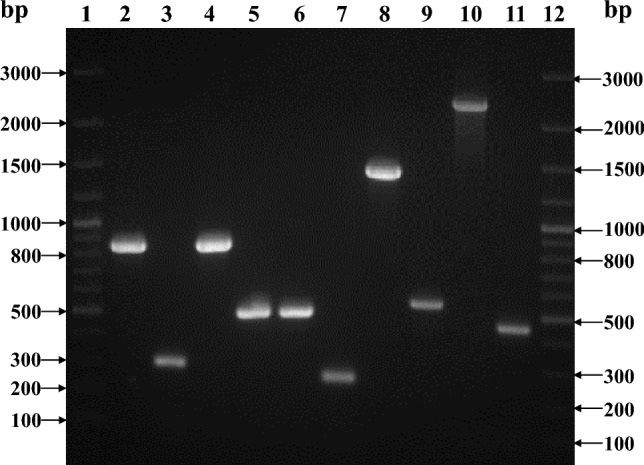
Agarose gel analysis of *msr*A, *msr*B, *msr*C, *msr*P and *bis*C gene deletions in *S.* Typhimurium. The deletions of above-mentioned genes were confirmed by PCR using test primers (as mentioned in Table 1) and genomic DNA from either *S*. Typhimurium (L2, L4, L6, L8, L10 respectively) or Δ5*msr* (L3, L5, L7, L9, L11 respectively) strain as templates. Lane 1 and Lane 12 are 100 bp DNA ladder.Sizes of PCR products are mentioned in the figure itself. The image displayed is cropped from the full-length gel. The full-length gel is presented in supplementary figure S1.

Deletions of the *msr*A, *msr*B, *msr*C, *msr*P, and *bis*C genes in *S.* Typhimurium were confirmed by PCR. The test primers designed in the flanking regions to the various genes yielded smaller amplicons in the mutant strains. However, bigger size PCR products were observed in *S.* Typhimurium (Fig. 1).

### Deletion of all the *msr*s extends initial growth in lag and log phases of *S.* Typhimurium

**Figure 2:**
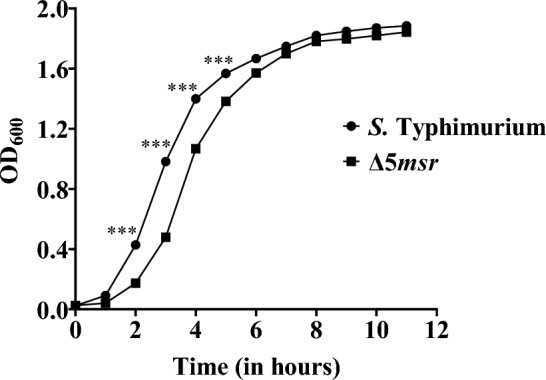
Δ5*msr* mutant strain shows extended lag and log phases of in vitro growth. The Δ5*msr* mutant and *S.* Typhimurium strains were grown in LB broth for 11 h. Aliquots were taken at hourly intervals and absorbance were recorded at 600 nm. Data are presented as mean ± SD (n = 3) and analyzed by two-way ANOVA. ***p < 0.001.

The growth of the Δ5*msr* and *S.* Typhimurium strains was assessed for a period of 11 h. Sigmoidal growth curves were observed for both strains. As compared to *S.* Typhimurium, the Δ5*msr* mutant strain showed a considerable lag in the growth phase for the initial six hours, however, both strains grew similarly from 7 h onwards (Fig. 2).

### Δ5*msr* mutant strain shows hypersusceptibility to oxidants in vitro

**Figure 3:**
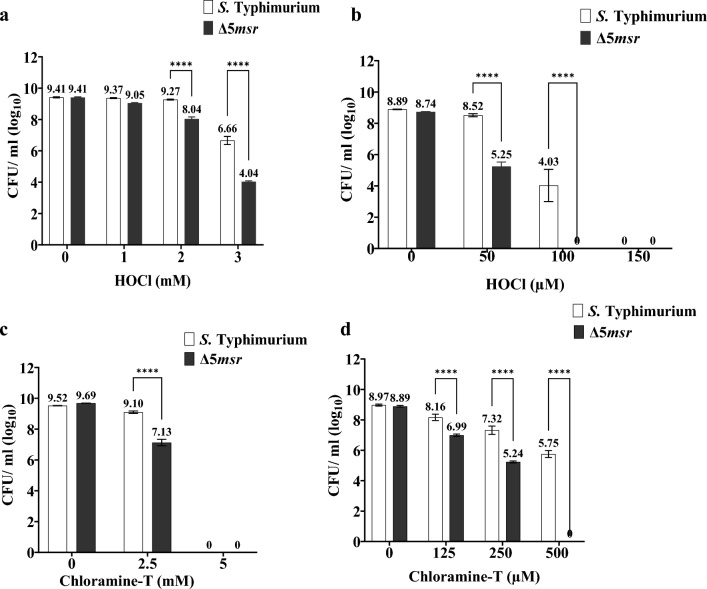
Δ5*msr* mutant is highly susceptible to HOCl and ChT. Δ5*msr* mutant and *S.* Typhimurium strains were grown up to mid-log phase in LB broth and incubated with the indicated concentrations of HOCl or ChT, either directly adding to culture media (**a**, **c**), or after pelleting and suspending cultures in PBS (**b**, **d**). Following incubation of two hours (**a**, **c**) or 30 min (**b**, **d**), the cultures were serially diluted and plated on agar media. CFUs were enumerated following overnight incubation of the plates. Data are presented as mean ± SE (n = 8) for (**a**), (n = 9) for (**b**), (n = 3) for (**c**) and (n = 8) for (**d**) Data were analyzed by two-way ANOVA. ****p < 0.0001.

Met residues are highly prone to oxidation. However, by repairing Met-SO, Msrs enhance the survival of bacterial pathogens under oxidative stress. We assessed the susceptibility of exponentially growing Δ5*msr* mutant strain in LB media to various oxidants in vitro*.* Compared to *S.* Typhimurium, the Δ5*msr* mutant strain was slightly (~ 2 ), but not significantly, susceptible to 1 mM HOCl. However, the mutant strain showed ~ 14-fold more susceptibility (*p* < 0.0001) to 2 mM HOCl. Furthermore, the mutant strain was extremely susceptible (~ 1200 fold more; *p* < 0.0001) to 3 mM HOCl (Fig. 3a).

Similarly, when the cultures were suspended in PBS and exposed to HOCl, the Δ5*msr* mutant strain was ~ 900-fold more susceptible to 50 µM HOCl than the *S.* Typhimurium strain. We did not observe any colonies following the exposure of the Δ5*msr* mutant strain to 100 µM HOCl. However, *S.* Typhimurium exposed to 100 µM HOCl showed (log_10_, mean ± SE) 4.03 ± 1.03 CFUs/ ml (Fig. 3b).

In comparison to *S.* Typhimurium, the Δ5*msr* mutant strain showed ~ 76 fold more susceptibility to 2.5 mM ChT (*p* < 0.0001) in LB media. However, both strains did not survive when exposed to 5 mM ChT (Fig. 3c). Interestingly, the Δ5*msr* mutant strain showed ~ 25 and ~ 300 fold more susceptibility than *S.* Typhimurium when cultures were suspended in PBS and exposed to 125 µM and 250 µM of ChT respectively (*p* < 0.0001). We did not recover any viable colonies following the incubation of the Δ5*msr* mutant strain with 500 µM ChT (Fig. 3d). However, the recovered numbers (log_10_, mean ± SE) in the case of *S.* Typhimurium exposed to 500 µM ChT were 5.75 ± 0.64 CFUs/ ml.

Surprisingly, in LB media both strains were not susceptible when exposed to 5% paraquat. However, the Δ5*msr* mutant strain was susceptible to 10% paraquat exposure, but it was not significantly different from that of *S.* Typhimurium.

**Figure 4:**
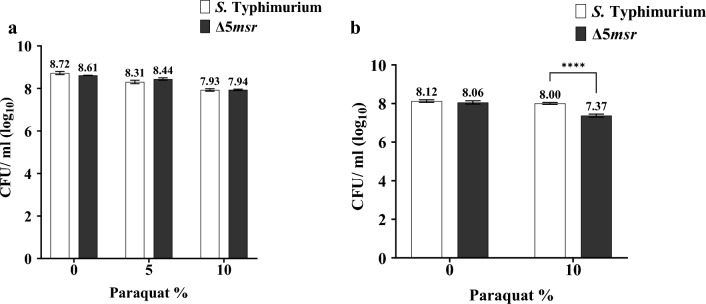
Δ5*msr* is highly susceptible to paraquat under non-growing conditions but not under growing conditions. Mid-log grown cultures of Δ5*msr* mutant and *S.* Typhimurium strains were incubated with the indicated concentrations of paraquat, either directly adding to culture media (**a**), or after pelleting and suspending cultures in PBS (**b**). Following 2 h of incubation, the cultures were serially diluted and plated on agar media. CFUs were enumerated following overnight incubation of the plates. Data are presented as mean ± SE (n = 3) for (**a**) and (n = 6) for (**b**) Data were analyzed using two-way ANOVA. ****p < 0.0001.

In separate experiments where the cultures were suspended in PBS and exposed to 10% paraquat, the Δ5*msr* mutant strain showed ~ fourfold more susceptibility (*p* < 0.0001) in comparison to *S.* Typhimurium (Fig. 4b). Taken together, our data suggest that the Msrs contribute to the survival of *S.* Typhimurium against oxidants.

### Δ5*msr* mutant strain depicts higher MDA levels

**Figure 5:**
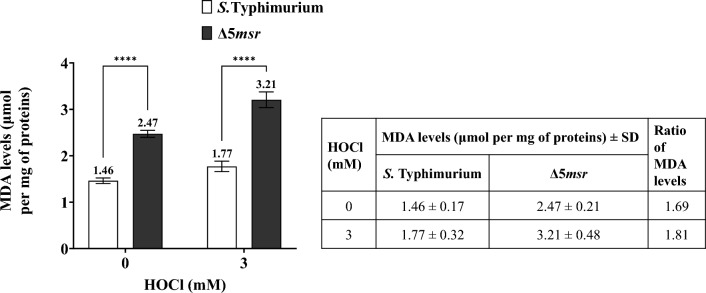
Δ5*msr* mutant strain depicts higher MDA levels. Cell-free lysates of 0 and 3 mM HOCl exposed cultures of Δ5*msr* mutant and *S.* Typhimurium strains were incubated with TBA. MDA levels were estimated as described in materials and methods. Ratios of MDA levels are depicted alongside figure. The data is presented as mean ± SE (n = 3) and analyzed by two-way ANOVA. ****p < 0.0001.

Lipids are highly prone to oxidation. MDA is a marker of lipid peroxidation^22^. We next assessed the levels of MDA in both strains. MDA levels (mean ± SE; micromoles per mg of proteins) in 0 mM HOCl exposed Δ5*msr* mutant and *S.* Typhimurium strains were 2.47 ± 0.21 and 1.46 ± 0.17 respectively. However, upon 3 mM HOCl exposure, the levels were 3.21 ± 0.48 and 1.77 ± 0.32 in Δ5*msr* mutant and *S.* Typhimurium strains respectively. The MDA levels were significantly higher (*p* < 0.0001) in the Δ5*msr* mutant strain than in *S.* Typhimurium (Fig. 5).

### Δ5*msr* mutant strain accumulates higher levels of protein carbonyls

**Figure 6:**
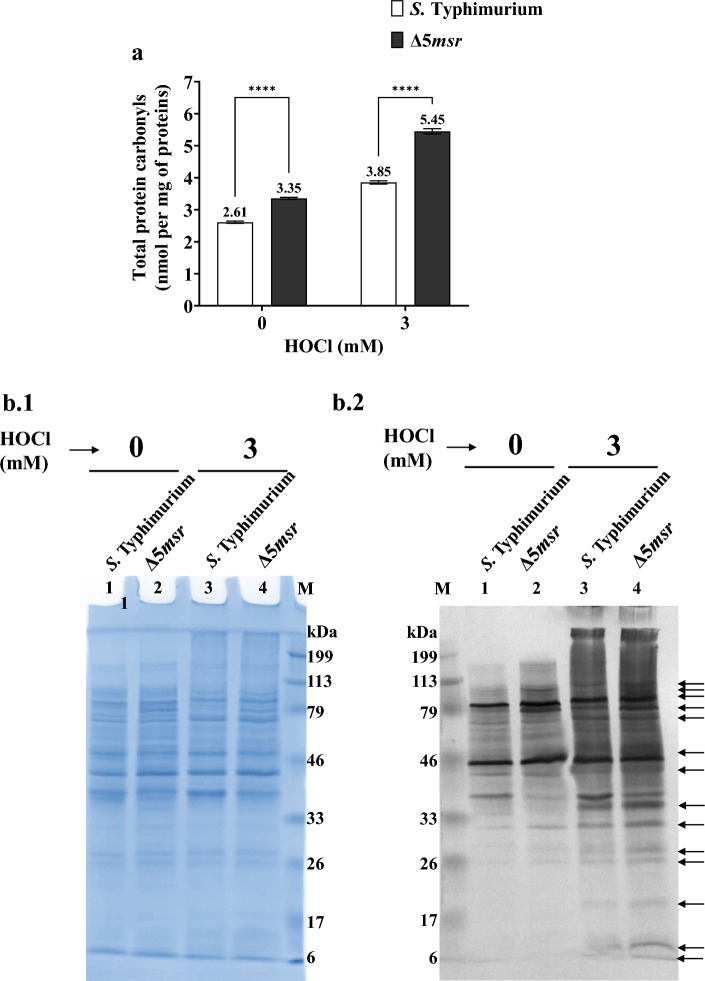
(**a**) Δ5*msr* mutant accumulates more protein carbonyls. Cell-free lysates of Δ5*msr* mutant and *S.* Typhimurium strains were derivatized with DNPH. Total protein carbonyl levels were estimated as described in methods. The data is presented as mean ± SE (n = 3) and analyzed by two-way ANOVA. ****p < 0.0001. (**b**) Oxyblot analysis of Δ5*msr* mutant and *S.* Typhimurium strains. Cell-free lysates of 0 mM and 3 mM HOCl exposed cultures of Δ5*msr* mutant and *S.* Typhimurium strains were derivatized with 2, 4-DNPH. 20 µg of proteins were resolved on SDS gels. Separated proteins were electroblotted to PVDF membrane. Following blocking, the membranes were incubated in anti-DNPH antibodies and secondary antibodies conjugated with alkaline phosphatase. Blots were developed using NBT and BCIP as substrates. Arrows indicate darker bands in Δ5*msr* lysate loaded lane (6 b.2). Coomassie stained gel served as loading control (6 b.1). The images displayed are cropped from the full-length gels. The full-length gels are presented in supplementary figures S1 (6 b.1) & S2 (6 b.2). ImageJ analysis of the blots is presented in supplementary Table T1.

Carbonyls are considered stable markers of protein oxidation. We next determined the levels of total protein carbonyls in the Δ5*msr* mutant and *S.* Typhimurium strains. The carbonyls were assessed spectrophotometrically as well as by oxyblot. Total protein carbonyl levels (mean ± SE; per mg of proteins) were 3.35 ± 0.21 and 2.61 ± 0.15 in Δ5*msr* mutant and *S.* Typhimurium strains, respectively. The carbonyl levels following exposure to 3 mM HOCl were 5.45 ± 0.15 and 3.85 ± 0.18 in Δ5*msr* mutant and *S.* Typhimurium strains respectively (Fig. 6a). The levels were significantly (*p* < 0.0001) higher in the mutant strain.

In oxyblot analysis, several bands (as indicated by arrows) were darker in Δ5*msr* mutant strain-loaded lanes as compared to the lanes loaded with *S.* Typhimurium lysates. Quantitation of the integrated densities of bands using ImageJ software also revealed darker areas in Δ5*msr* mutant strain-loaded lanes (Fig. 6b.2 and Supplementary Table T1).

### Δ5*msr* mutant strain shows less free amines

**Figure 7:**
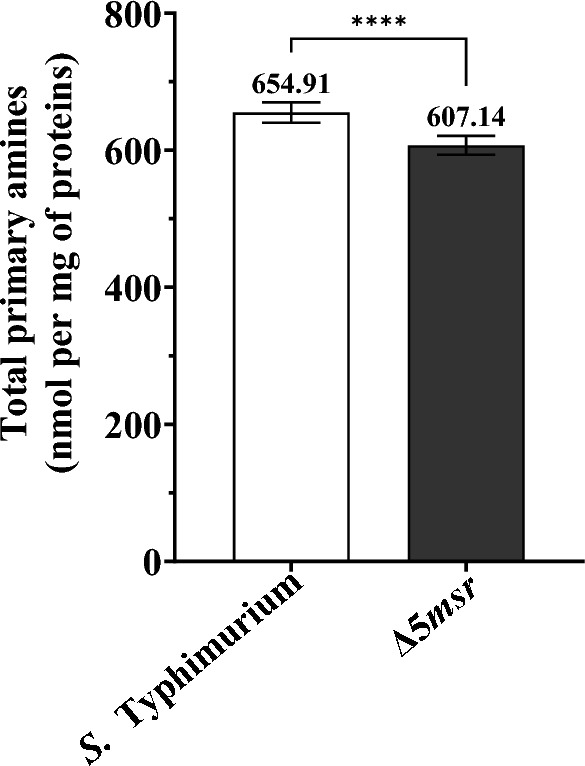
Δ5*msr* mutant depicts fewer primary amines. The lysates of mid-log grown cultures of Δ5*msr* mutant and *S.* Typhimurium strains were incubated with 0.01% TNBS. Total free amines were calculated as described in methods. The data is presented as mean ± SE (n = 18) and analyzed by students’ *t*-test. ****p < 0.0001.

Higher levels of ROS are associated with lower levels of free amines^23^. We estimated the free amine levels in the lysates of the Δ5*msr* mutant strain and *S.* Typhimurium. The Δ5*msr* mutant strain showed 1.3-fold (*p* < 0.0001) less free amines than *S.* Typhimurium (Fig. 7).

### Δ5*msr* mutant strain accumulates higher orders of oligomers:

**Figure 8:**
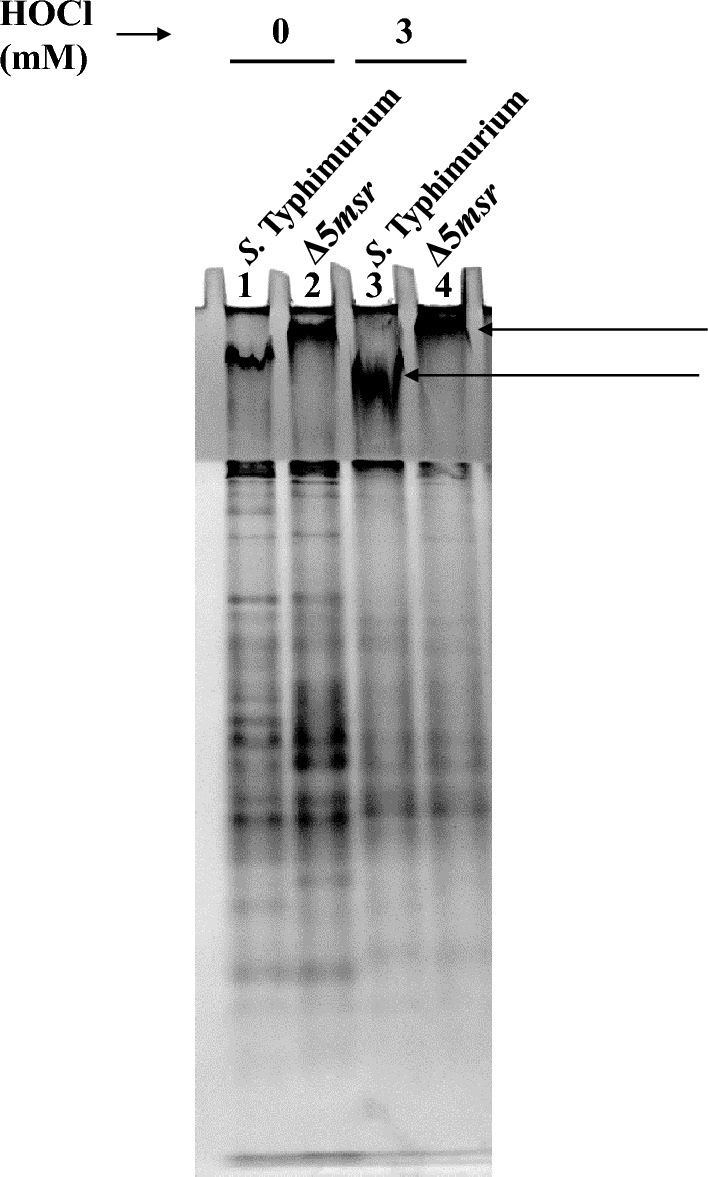
Δ5*msr* shows higher order multimers. Δ5*msr* mutant and *S.* Typhimurium strains were grown up to mid-log phase and exposed to indicated concentrations of HOCl. Following 2 h of exposure, the cultures were lysed. Cell-free lysates were resolved on native-PAGE. Arrows show migration of higher order multimers. The image displayed is cropped from the full-length gel. The full-length gel is presented in supplementary figure S4.

Met-oxidation is linked with oligomerization of the proteins^24^. Native gel analysis revealed varying sizes of protein oligomers. These oligomers were retained in the stacking gels in both strains. However, the degree of migration of higher-order oligomers was much slower in the Δ5*msr* mutant strain (Fig. 8, lanes 2 and 4) than in *S.* Typhimurium (Fig. 8, lanes 1 and 3). This suggests that Δ5*msr* strain accumulates oligomers of much higher molecular weight.

### Δ5*msr* mutant exhibits increased neutrophil sensitivity

**Figure 9:**
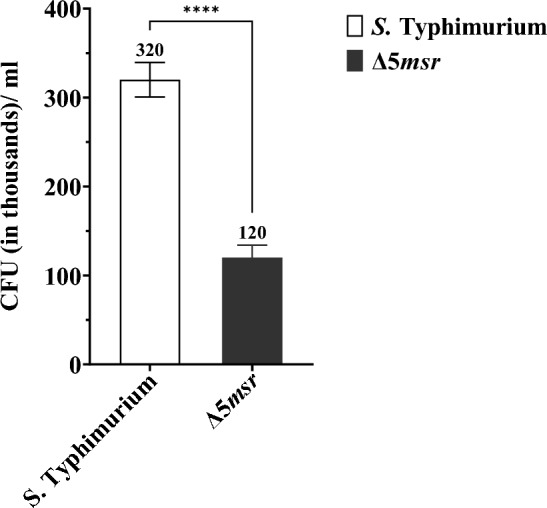
Δ5*msr* mutant strain shows high susceptibility to neutrophils. Δ5*msr* mutant strain and *S.* Typhimurium were incubated with neutrophils. Following incubation, the mix was centrifuged and neutrophils were lysed with 0.1% TritonX-100. The lysates were then serially diluted and plated on agar media. CFUs were counted following incubation of the plates. The data is presented as mean ± SE (n = 3) and analyzed by students’ *t*-test. ****p < 0.0001.

The role of *msrs* in survival against neutrophil-mediated killing was determined. The Δ5*msr* mutant and *S.* Typhimurium strains were incubated with neutrophils. Recovery of Δ5*msr* mutant and *S.* Typhimurium strains (mean CFUs/ ml; thousands ± SE) after 30 min of incubation were 120 ± 47 and 320 ± 35 respectively. The Δ5*msr* mutant strain showed 2.67 fold more (*p* < 0.0001) susceptibility to neutrophils (Fig. 9). Treatment with Triton X-100 had no significant effects on bacterial counts (Supplementary Fig. S5).

### Δ5*msr* mutant strain shows defective survival in mice spleen and liver

Δ*msr*A, Δ*msr*P, Δ*bis*C, Δ*msr*AΔ*msr*B, Δ*msr*AΔ*msr*C and Δ*msr*AΔ*msr*B mutants depicted mild to moderate defect in colonization in mice tissues^16,19^. We assessed the effect of deletions of all *msrs* on the virulence of *S.* Typhimurium in mice. The Δ5*msr* mutant strain has been highly defective (*p* < 0.0001) in colonization in mice spleen and liver (Table 1).

**Table 1:** Δ5*msr* mutant strain shows defective fitness in mice spleen and liver.

Days post infection	CI (mean ± SE)	
	Spleen	Liver
3	0.032 ± 0.017 (n = 8)	0.016 ± 0.009 (n = 8)
5	0.111 ± 0.088 (n = 8)	0.018 ± 0.012 (n = 8)

The mice were inoculated with a mixture of Δ5*msr* mutant and *S.* Typhimurium strains (in a ratio of 1: 1). Mice (at each time point, n = 8) were dissected on days 3 and 5. Bacterial burdens of Δ5*msr* mutant and *S.* Typhimurium strains were determined in spleen and liver and CIs were calculated as described in materials and methods.

## Discussion

Survival of *Salmonella* inside the host, at least in part, depends upon its ability to quench oxidants generated by the host's inflammatory response. Primary antioxidant enzymes play a crucial role in defending host-generated oxidants^14,25–28^. The scavenging abilities of SODs, catalases, etc., generally fall short of the quantity of oxidants generated during a respiratory burst. This leads to macromolecular damage. Owing to location and reactivity, proteins (Met residues in particular) are highly prone to oxidation which leads to compromised protein function. The functional protein pool in the cell can be maintained by protease-mediated degradation of oxidized proteins followed by ribosomal synthesis^29^. However, Msr-mediated repair is energetically cheaper and a relatively rapid way to maintain an active protein pool in the cell. This not only helps to thrive bacterial pathogens under stress conditions but might also aid in the infection process. Therefore, Msrs might contribute to the virulence of bacterial pathogens including *S.* Typhimurium. Indeed, *msr* gene deletion strains of several bacterial pathogens showed defective survival under oxidative stress conditions^15,16,18,19,30,31^ and attenuated virulence^10,16,21,32,33^. As stated above, *S.* Typhimurium encodes five *msrs.* The above studies assessed the roles of single or two Msrs together in the stress survival of *S.* Typhimurium. To determine the importance of Met-SO repair in whole cell physiology and stress survival of *S.* Typhimurium, first, we deleted all five known *msrs* from *S.* Typhimurium. The pan *msr* deletion (Δ5*msr*) strain might not be able to repair Met-SO. The deletions were confirmed by PCR. The primers localized in flanking regions to target genes amplified bigger size amplicon in *S.* Typhimurium while smaller in Δ5*msr* mutant strain (Fig. 1). The Δ5*msr* mutant strain showed a slightly reduced growth in the lag and early log phases as compared to *S.* Typhimurium (Fig. 2). O_2_^−^ generated due to leaky respiratory chain and subsequently other ROS formed might have oxidized Met residues, and due to lack of active repair, the Δ5*msr* mutant strain showed moderate in vitro growth defect.

ROS/ RNS-mediated oxidation of bacterial macromolecules is the primary way by which phagocytic cells kill invading bacterial pathogens. *Salmonella* must have evolved the mechanism(s) to counter ROS/ RNS-mediated damage. Primary antioxidants and protein repair enzymes are a few important mechanisms employed by *Salmonella* to survive against ROS/ RNS-mediated damage. Msr mediated repair of Met-SO acts as a dual-edged sword. First, Msr restores the function of Met-SO containing proteins^10^. Second, Met residues act as a sink for oxidants^9^. Cyclic oxidation and reduction of Met (free or in proteins) residues reduces overall cellular oxidant levels^34^. We hypothesized that deletion of all *msrs* might render *S.* Typhimurium highly susceptible to oxidants. In comparison to *S.* Typhimurium, the Δ5*msr* mutant strain has been manyfold susceptible to HOCl (*p* < 0.0001) and ChT (*p* < 0.0001) under growing and non-growing conditions (Fig. 3), but to paraquat (*p* < 0.0001) under non-growing condition (Fig. 4). Interestingly, neither of the strains exhibited sensitivity to 5% paraquat exposure under growing conditions. Although the Δ5*msr* mutant and *S.* Typhimurium strains exhibited slight sensitivity to 10% paraquat under growing conditions, however, there was no significant difference between the sensitivities of the two strains. This could be attributed to the oxidant-quenching ability of the growth medium ^35^. Furthermore, the Δ5*msr* mutant strain expresses higher levels of SODs, potentially aiding the mitigation of superoxide radicals generated upon the addition of paraquat. Consequently, higher paraquat concentrations might be necessary to render the Δ5*msr* mutant strain susceptible to its oxidative effects. In a previous study, the Δ*msr*P strain of *S.* Typhimurium showed mild sensitivity (about 7.7 fold) to 100 µM HOCl exposure^21^. However, the Δ*msr*A and Δ*msr*AΔ*msr*C strains of *S.* Typhimurium displayed significantly higher susceptibilities (~ 93 fold and ~ 3000 fold respectively) to the same concentration of HOCl^19^. Likewise, in our study, the Δ5*msr* mutant strain showed extreme sensitivity to HOCl. Notably, no viable colonies were observed following exposure of the Δ5*msr* mutant strain to 100 µM HOCl (Fig. 3b).

During the respiratory burst, taurine gets oxidized by HOCl and converted into chlorotaurine. Chlorotaurine is a relatively mild but stable oxidant that acts as a sustained source of oxidative stress^36,37^. ChT is a synthetic analog of chlorotaurine which preferentially oxidizes Met residues^38^. The Δ*msr*P strain of *S.* Typhimurium showed similar sensitivity to 200 µM ChT exposure^21^. However, the Δ*msr*A and Δ*msr*AΔ*msr*C strains have been ~ 49 and ~ 112 fold more susceptible to 200 µM ChT (our unpublished observations). Interestingly, the Δ5*msr* mutant strain showed ~ 1600 fold more sensitivity to 250 µM ChT as compared to *S.* Typhimurium (Fig. 4a). These observations suggest that while each Msr contributes to *S.* Typhimurium in resisting oxidative stress, the cumulative effects of deletion of all Msrs renders *S.* Typhimurium extremely sensitive to oxidants.

Lipids are highly susceptible to ROS-mediated oxidation. We sought to explore if deletion of *msrs* increases lipid peroxidation in *S.* Typhimurium. MDA is a well-known marker of lipid peroxidation^22^. Indeed, higher MDA levels (*p* < 0.0001) were observed in Δ5*msr* mutant strain as compared to *S.* Typhimurium, which further increased (*p* < 0.0001) upon HOCl exposure (Fig. 5). This further substantiates that Msrs play an important role in maintaining ROS homeostasis and resisting oxidative stress in *S.* Typhimurium.

Carbonyls are considered as stable markers of protein oxidation^11^. Therefore, carbonyls have been emphasized as hallmarks of oxidative stress^39^. We investigated whether the deletion of *msrs* affects carbonyl levels. Higher carbonyl levels (*p* < 0.0001) were observed in the Δ5*msr* mutant strain as compared to *S.* Typhimurium (Fig. 6).

Levels of free amines are inversely proportional to protein oxidation^23^. We observed lower levels of free amines in the Δ5*msr* mutant strain as compared to *S.* Typhimurium (*p* < 0.0001, Fig. 7).

Met oxidation can change the surface hydrophobicity of the proteins^40^, causing exposure of the buried hydrophobic residues which leads to unfolding and aggregation^41^. Oxidation of purified malate synthase of *S.* Typhimurium^10^, catalase of *H. pylori*^24^ and the *E. coli* chaperone GroEL^42^ leads to their aggregation. We explored the effect of deletion of all *msrs* on protein aggregation in *S.* Typhimurium. Native gel analysis suggests the accumulation of higher molecular weight oligomers in the Δ5*msr* mutant strain (Fig. 8).

Neutrophils play a very important role in host defense against *Salmonella* infection^43^. Due to the presence of NOX (source of superoxide ion) and MPO (source of HOCl) systems, the neutrophils produce copious amounts of ROS and RCS^44^, which directly kill the invading bacteria. *Salmonella* has evolved several strategies to survive neutrophil attack^45,46^. One such strategy is Msr mediated repair of Met-SO. The MsrP plays a minimal role in the survival of *S.* Typhimurium against neutrophils^21^. On the other hand, the Δ*msr*A^18^ and Δ*msr*AΔ*msr*C strains (our unpublished observations) of *S.* Typhimurium showed hypersensitivity to neutrophils. Our findings indicate that the Δ5*msr* mutant strain is significantly more (*p* < 0.0001) sensitive to neutrophils as compared to *S.* Typhimurium (Fig. 9). Furthermore, the observed sensitivity was a direct result of the interaction of neutrophils with the bacteria and the addition of Triton X-100 did not affect CFU/ ml of both the strains (Supplementary Fig. S5), suggesting that deletion of *msrs* doesn’t enhance the sensitivity of *S.* Typhimurium to 0.1% Triton X-100.

The contributions of Msrs in the virulence of *S.* Typhimurium have been assessed by using single or double gene deletion strains^16,17,21^. The Δ*msr*A and Δ*bis*C mutant strains of *S.* Typhimurium exhibited fitness defects in the spleen and liver of mice. The CI for Δ*msr*A mutant in mice spleen and liver were 0.24 ± 0.07 and 0.16 ± 0.03 respectively^16^. Similarly, the CI for Δ*bis*C mutant in mice spleen and liver were 0.2 and 0.16 respectively^17^. However, the Δ*msr*P strain of *S.* Typhimurium was moderately defective in mice tissues^21^. Whereas the Δ*msr*B and Δ*msr*C mutant strains of *S.* Typhimurium were not attenuated in mice tissues^16^.

In other studies, double gene deletion mutants of *S.* Typhimurium were generated to investigate the additive effect of *msr* deletions on the virulence of *S.* Typhimurium. The Δ*msr*AΔ*msr*B and Δ*msr*AΔ*msr*C strains were highly defective in fitness in mice spleen and liver. The CI values of Δ*msr*AΔ*msr*B^16^ and Δ*msr*AΔ*msr*C (our unpublished observations) strains were 0.13 ± 0.05 and 0.21 ± 0.07 respectively in mice spleen, and 0.23 ± 0.09 and 0.11 ± 0.02 respectively in mice liver. However, the Δ*msr*BΔ*msr*C double mutant strain showed a moderate defect in mice tissues. The Δ5*msr* mutant strain exhibited severe defects in the spleen and liver of mice. CI values in mice spleen and liver were 0.032 ± 0.017 and 0.016 ± 0.009 respectively on day 3 post-infection (Table 1). The lower CI values of the Δ5*msr* mutant strain (in mice tissues) as compared to single or double *msr* deletion strains is likely due to the combined effects of deletion of all five Msrs. These findings highlight the essential roles of Msrs in *Salmonella* survival and virulence in the host.

By repairing Met-SO to Met, Msrs maintain overall ROS homeostasis in cells and prevent ROS-mediated macromolecular damage. Our data suggests that Msrs not only contribute to the survival of *S.* Typhimurium against oxidative stress but also aid in the colonization of this bacterium in animal tissues. Earlier studies suggested mild to moderate attenuation of single/ double *msr* deletion(s) strains. The highly attenuated phenotype observed in the current study might be due to the combined effect of deletions of all *msrs.* The current study might pave the way to develop novel therapeutic/ prophylactic strategies against this bacterium of zoonotic importance.

## Methods

### Construction and confirmation of pan *msr *gene deletion strain (Δ5*msr*) in *S.* Typhimurium

The Δ*msr*AΔ*msr*C mutant strain (available in our lab) was revived and confirmed by PCR^19^. Further deletions of *msr*P, *msr*B and *bis*C genes were carried out by the 1-step gene inactivation method^47^. Briefly, the plasmid pKD46 was introduced into the Δ*msr*AΔ*msr*C strain^19^ by electroporation. The antibiotic cassette along with homologous regions to the flanking genes were amplified from plasmid pKD3 using primers as detailed in Table 2. The cassette was purified and electroporated to λ-red recombinase expressing Δ*msr*AΔ*msr*C strain. The positive recombinants were selected on antibiotic containing media. Following PCR based screening, the antibiotic cassette was removed by pCP20 encoded FLP recombinase. All three genes were deleted one by one by similar method. All images were visualized using MultiImage™ Light Cabinet (Alpha Innotech Corp.) and acquired using AlphaImager EC software 3.2.2.

**Table 2:** Primers and PCR conditions used for deletion and confirmation of various genes in *S.* Typhimurium LT2 (NCBI accession no. NC_003197.2).

Serial No	Primer Name	Sequence	Binding location of primer in genome of *S.* Typhimurium LT2	Amplicon size	Purpose	PCR conditions	References
1	MsrA deletion Test Forward	5′ AGATACATTAATGTTGTTATT 3′	4,645,918 to 4,645,938	300 bp in Δ5*msr* and 850 bp in *S*. Typhimurium	Confirmation of *msr*A deletion	95 °C 5 min 95 °C 30 s 48 °C 40 s × 35 72 °C 90 s 72 °C 10 min	^19^
2	MsrA deletion Test Reverse	5′ GTAACGTTTAATGAAAACCG 3′	4,645,089 to 4,645,108				
3	MsrB deletion Forward	5′AGTTGGTACTGAGGTGTTAATGTTTTGTTAGAATCGGTCAGGCAATGTAGGCTG 3′	–	1096 bp	To amplify FRT flanked Chloramphenicol cassette	95 °C 5 min 95 °C 30 s 42 °C 30 s × 35 72 °C 90 s 72 °C 10 min	Current study
4	MsrB deletion Reverse	5′CATGATGTGTCCCCTCCTGTGGAATAATTTGCTGAATCGTTTTTTATGAATA 3′	–				
5	MsrB deletion Test Forward	5′ ATTCGCCTCACTCTTCCTTTCG 3′	1,369,809 to 1,369,830	498 bp in Δ5*msr* and 869 bp in *S*. Typhimurium	Confirmation of *msr*B deletion	95 °C 5 min 95 °C 30 s 62 °C 40 s × 40 72 °C 100 s 72 °C 10 min	Current study
6	MsrB deletion Test Reverse	5′ CCATCTGACCGTGAGTATCGA 3′	1,370,657 to 1,370,677				
7	MsrC deletion Test Forward	5′ GAGCAAGAACGCATTTAATGC 3′	1,944,530 to 1,944,550	280 bp in Δ5*msr* and 500 bp in *S*. Typhimurium	Confirmation of *msr*C deletion	95 °C 5 min 95 °C 30 s 48 °C 40 s × 40 72 °C 90 s 72 °C 10 min	^19^
8	MsrC deletion Test Reverse	5′ GCGTACGCAGGCCGTGTT 3′	1,944,054 to 1,944,071				
9	MsrP deletion Forward	5′GACCGGGAGTCTGTGATGAAAAAGATACGTCCATTAACAGAAGCCGTGTAGGCTGGAGCTGCTTC 3′	–	1092 bp	To amplify FRT flanked Chloramphenicol cassette	95 °C 5 min 95 °C 30 s 62 °C 40 s × 35 72 °C 100 s 72 °C 10 min	^21^
10	MsrP deletion Reverse	5′TGCTGTCAGACGCACTTAAAAATTCTCCCGCAAATTGAGACCGCGCATATGAATATCCTCCTTAG 3′	–				
11	MsrP ST test Forward	5′AGGGCCGTACGCTGGTGAAGAT 3′	3,547,964 to 3,547,985	550 bp in Δ5*msr* and 1401 bp in *S*. Typhimurium	Confirmation of *msr*P deletion	95 °C 5 min 95 °C 30 s 62 °C 40 s × 40 72 °C 100 s 72 °C 10 min	^21^
12	MsrP ST test Reverse	5′GAAACACCATAATCCTAACAGGCG 3′	3,549,341 to 3,549,364				
13	BisC deletion Forward	5′CTCCCTGCAAACCGTTGTGCATGACCAGGTGCACAGTAAAACGCGGTAGGTGG 3′	–	1103 bp	To amplify FRT flanked Chloramphenicol cassette	95 °C 5 min 95 °C 30 s 42 °C 30 s × 35 72 °C 90 s 72 °C 10 min	Current study
4	BisC deletion Reverse	5′CGCCTGCGGTAGGTTCAGGGTCCGGCCATGCGCCTTCGTGAATACAATGAATAT 3′	–				
15	BisC ST test Forward	5′TTGACCCACGCACCATCGCGT 3′	3,832,645 to 3,832,665	445 bp in Δ5*msr* and 2335 bp in *S*. Typhimurium	To amplify FRT flanked Chloramphenicol cassette	95 °C 5 min 95 °C 30 s 64 °C 40 s × 40 72 °C 150 s 72 °C 10 min	Current study
16	BisC ST test Reverse	5′TTATGAGTTGGCAGGCGGATCAA 3′	3,830,332 to 3,830,354				

### In vitro growth analysis

The in vitro growths of the Δ5*msr* mutant and *S.* Typhimurium strains were analyzed as described previously^48^. Isolated colonies were inoculated in 10 ml of LB broth. Following overnight incubation, the cultures were diluted 1: 100 in fresh medium. The cultures were then grown in a shaker incubator at 37 °C at 180 rpm. The optical densities (OD) were measured at 600 nm at an interval of 1 h.

### In vitro oxidant susceptibility assays

Overnight grown cultures of the Δ5*msr* mutant and *S.* Typhimurium strains were diluted in LB broth (at 1: 100 ratio) and incubated at 37 °C in a shaker incubator to an OD_600_ of 0.8. The cultures were then exposed to various concentrations of HOCl (NaOCl, Sigma), chloramine T (ChT) (SRL, India), or methyl viologen (paraquat, Sigma) for 2 h. In parallel experiments, the mid-log phase grown cultures were pelleted, washed and suspended in PBS to an OD_600_ of 1.0. The suspensions were then exposed to various concentrations of HOCl, ChT, or paraquat. Following 30 min (for HOCl and ChT) or 2 h (for paraquat) of incubation at room temperature in the dark, the excess oxidants were quenched by the addition of L-methionine (10 mM final) for 15 min. Following incubation, the suspensions were ten-fold serially diluted and plated on agar media. CFUs/ ml were calculated after overnight incubation of the plates at 37 °C.

### Preparation of cell-free lysates

Mid-log phase grown cultures of Δ5*msr* mutant or *S.* Typhimurium strains were exposed to PBS or HOCl, harvested and washed twice with PBS by centrifugation at 4500×*g* at 4 °C for 10 min. Pellets were lysed by sonication. Unbroken cells and debris were removed by centrifugation at 15,000×*g* for 30 min at 4 °C. Supernatants were collected, aliquoted and stored at − 80 °C. Total proteins in such lysates were estimated using the Pierce™ BCA Protein Assay Kit (Thermo Scientific).

### Quantitation of malondialdehyde (MDA) levels

MDA levels were estimated using TBARS assays as described elsewhere^49^ with minor modifications. Cell-free lysates of Δ5*msr* mutant and *S.* Typhimurium strains were diluted to a concentration of 5 mg/ml. 500 µg of proteins (in 100 µl) were incubated with an equal volume of 10% TCA prepared in 0.25 N HCl (5% TCA final concentration) for 5 min. The mixtures were centrifuged at 15,000×*g* for 10 min, supernatants were collected and incubated with 100 µl of 0.67% TBA (0. 375% final concentration) for 10 min in a boiling water bath. Absorbance was recorded at 535 nm against a blank containing all the reagents except the sample. MDA levels were calculated using the formula c = A_535_/(*ε*l), where *ε* = 1.56 × 10^5^ M^−1^ cm^–1^ (molar absorption coefficient of TBA-MDA abduct is 1.56 × 10^5^ M^−1^ cm^−1^), c is the concentration of MDA in moles/ liter (M), and A = absorbance at 535 nm and expressed as micromoles per mg proteins^50^.

### Estimation of primary amines

Primary amines were estimated by TNBSA assays, as described previously (Habeeb, 1966) with minor modifications. Briefly, cell-free lysates were diluted with 0.1 M sodium bicarbonate (pH 8.5) to a concentration of 100 µg/ ml of proteins. Fifty micrograms of samples were incubated with 0.25 ml of freshly prepared 0.01% TNBS (Sigma) in 0.1 M sodium bicarbonate (pH 8.5) for 2 h at 37 °C. The reaction was stopped by the addition of 0.25 ml of 10% SDS and 0.125 ml of 1 N HCl. Absorbance was recorded at 335 nm. Primary amines in the lysates were quantified using a standard curve generated using L-methionine standards.

### Estimation of total protein carbonyls

Total protein carbonyls were estimated using 2, 4 dinitrophenyl hydrazine (2, 4 DNPH) assays^51^. 150 µl of cell-free lysates (a total of 750 µg of proteins) were incubated with 600 µl of 2, 4 DNPH solution (10 mM in 2.5 M HCl) for an hour in the dark. The mixtures were briefly vortexed every 15 min. Proteins were recovered by the addition of 750 µl of TCA (10% final concentration) on ice and centrifugation at 15,000×*g* for 20 min at 4 °C. The pellets were then washed twice with 10% TCA. Free DNPH was removed by washing with ethanol and ethyl acetate (1: 1). The final pellets were air-dried, dissolved in 6 M guanidine hydrochloride, and incubated at 37 °C for 30 min with intermittent vortexing. The absorbance was recorded at 355 nm using 6 M guanidine hydrochloride as a blank. Carbonyl levels were calculated using the formula c = A_355_/(*ε*l), where *ε* = 22,000 M^−1^ cm^−1^ (molar absorption coefficient of hydrazone is 22,000 M^−1^ cm^−1^), c is the concentration of carbonyl groups in moles/ liter (M), and A = absorbance at 355 nm^52^

### Oxyblot analysis

Oxyblot analysis was performed using an OxyBlot™ Protein Oxidation Detection kit (EMD Millipore), as described previously^51^. One hundred micrograms of cell-free lysates were denatured with SDS (6% final) and derivatized with 2, 4 DNPH for 15 min at room temperature. Following derivatization, the samples were incubated in a neutralization solution. 20 µg of derivatized proteins were resolved on 10% SDS gel and electroblotted onto a PVDF membrane. Free sites on the membrane were blocked by 2% skimmed milk prepared in PBS-T (0.05% Tween 20). After six washes with PBST, the membrane was incubated with anti-DNPH antibodies (1: 150 dilution) for 3 h at 37 °C. After washing, the membrane was washed with anti-rabbit goat IgG conjugated with alkaline phosphatase (1: 15,000; Sigma) for 4 h at 37 °C. NBT and BCIP were used as substrates for the development of the blots^51^. Gels and their blots were visualized in the Bio-Rad GelDoc™ imaging system and images were acquired using the Image Lab software ver. 3.0. Quantification of the densities of the bands in various lanes was done using ImageJ software.

### Native polyacrylamide gel electrophoresis

Accumulation of protein aggregates was determined using native polyacrylamide gel electrophoresis^10^. 100 µg of cell-free lysates were resolved in native gels (4% stacking, 8% separating). The gels were stained with CBB-R250 and visualized using the Bio-Rad GelDoc™ imaging system and images of the gels were acquired using the Image Lab software ver. 3.0.

### Neutrophil sensitivity assays

The sensitivity of Δ5*msr* mutant and *S.* Typhimurium strains to neutrophils was evaluated as previously described^18^ with minor modifications. Briefly, anticoagulant mixed goat blood was diluted with sterile PBS in 1: 1 ratio. A double-density gradient was prepared by layering equal volumes of Histopaque 1077 over Histopaque 1119 (Sigma). The diluted blood was layered over the Histopaque gradient. Following centrifugation at 750×*g* for 45 min at room temperature, the neutrophils were collected from the lower Histopaque 1077/ 1119 interface. The cells were washed twice with RPMI-1640 (complete, without phenol red) at 250×*g* for 10 min, resuspended in the same medium and kept on ice. The total number of viable cells was counted by the trypan blue dye exclusion method. Additionally, morphological identification of neutrophils was done by Giemsa staining. Neutrophils were adjusted to 2 × 10^6^ cells/ ml. 250 µl of cells (0.5 × 10^6^ cells) per well were seeded into 24-well tissue culture plates. Mid-log phase grown cultures of Δ5*msr* mutant and *S.* Typhimurium strains were pelleted, washed and resuspended in RPMI-1640 medium. Neutrophils and bacterial cultures were mixed in a multiplicity of infection (MOI) of 1: 1 (neutrophils: bacteria). The mix was then incubated in a CO_2_ incubator at 37 °C for 30 min. Following incubation, the mix was centrifuged at 13,000 rpm for 3 min. The supernatant was discarded and the neutrophils were lysed by 0.1 percent Triton X-100 for 5 min. In a different experiment, neutrophil isolation was excluded and only the resuspended cultures of Δ5*msr* mutant and *S.* Typhimurium strains (~ 0.5 × 10^6^ cells) were incubated with either 0.1 percent Triton X-100 or PBS for 5 min. The detergent action of Triton X-100 was neutralized by the addition of an equal volume of 1 X PBS to the mix. Following this, lysates were tenfold serially diluted and plated on agar plates. The colonies were counted following overnight incubation of the plates.

### Assessment of virulence in mice

Animal experiments were conducted with the approval of the Institutional Animal Ethics Committee (IAEC) at the Indian Council of Agricultural Research-Indian Veterinary Research Institute (ICAR-IVRI), Izatnagar, India, under the approval file No. F.26–1/2022–23/J.D.(R)/IAEC MEETING. All animal procedures strictly adhered to the guidelines and regulations set forth by IAEC, ICAR-IVRI, Izatnagar, India. The experimental protocols were conducted in accordance with the ARRIVE guidelines. The effect of deletion of all *msr* genes in the virulence of *S.* Typhimurium was evaluated in a mouse model^16,48^. In brief, six to seven weeks old Swiss Albino mice were intraperitoneally infected with a mixture of the Δ5*msr* strain carrying a chloramphenicol cassette (Δ5*msr*:: Cm) and *S.* Typhimurium strain (8 × 10^4^ bacteria in 100 µl in 1: 1 ratio). The actual inoculated numbers were determined by retrospective plating. Eight mice were sacrificed on days 3 and 5 post-infection, and their spleens and livers were harvested. The whole spleen and 100 mg of liver tissues were homogenized in PBS, and the homogenates were tenfold serially diluted and plated on HE agar as well as HE agar supplemented with chloramphenicol. The colonies observed on chloramphenicol-supplemented with HE agar plates were considered Δ5*msr* mutant strain colonies. The number of *S.* Typhimurium colonies was calculated by subtracting the number of colonies that appeared on chloramphenicol-supplemented HE agar plates from the total number of colonies that appeared on unsupplemented HE agar plates. Competitive indices (CI) were calculated as described previously^16,48^. The CI is the ratio of the number of Δ5*msr* strain CFU to *S.* Typhimurium CFU recovered divided by the ratio of the numbers of Δ5*msr* CFU strain to *S.* Typhimurium CFU inoculated.

### Statistical analysis

Data were analyzed by GraphPad Prism version 9.2 (Trial). Comparisons between multiple groups were done using either two-way analysis of variance (ANOVA) followed by Tukey’s post-hoc test or students’ *t-*test. *p* < 0.01 was considered significant among different test groups.

## Data availability

The data generated and analyzed in the current study are available with the corresponding author.

### Supplementary Information

Supplementary Information.
