# Supplementary material for: Pan msr gene deleted strain of Salmonella Typhimurium suffers oxidative stress, depicts macromolecular damage and attenuated virulence
Source: Sci Rep. 2023 Dec 9;13:21852. doi: 10.1038/s41598-023-48734-w (PMC10710478; doi:10.1038/s41598-023-48734-w)
Supplement: Supplementary file 1 — Supplementary Information. [file 41598_2023_48734_MOESM1_ESM.pdf]

Supplementary Figure S1

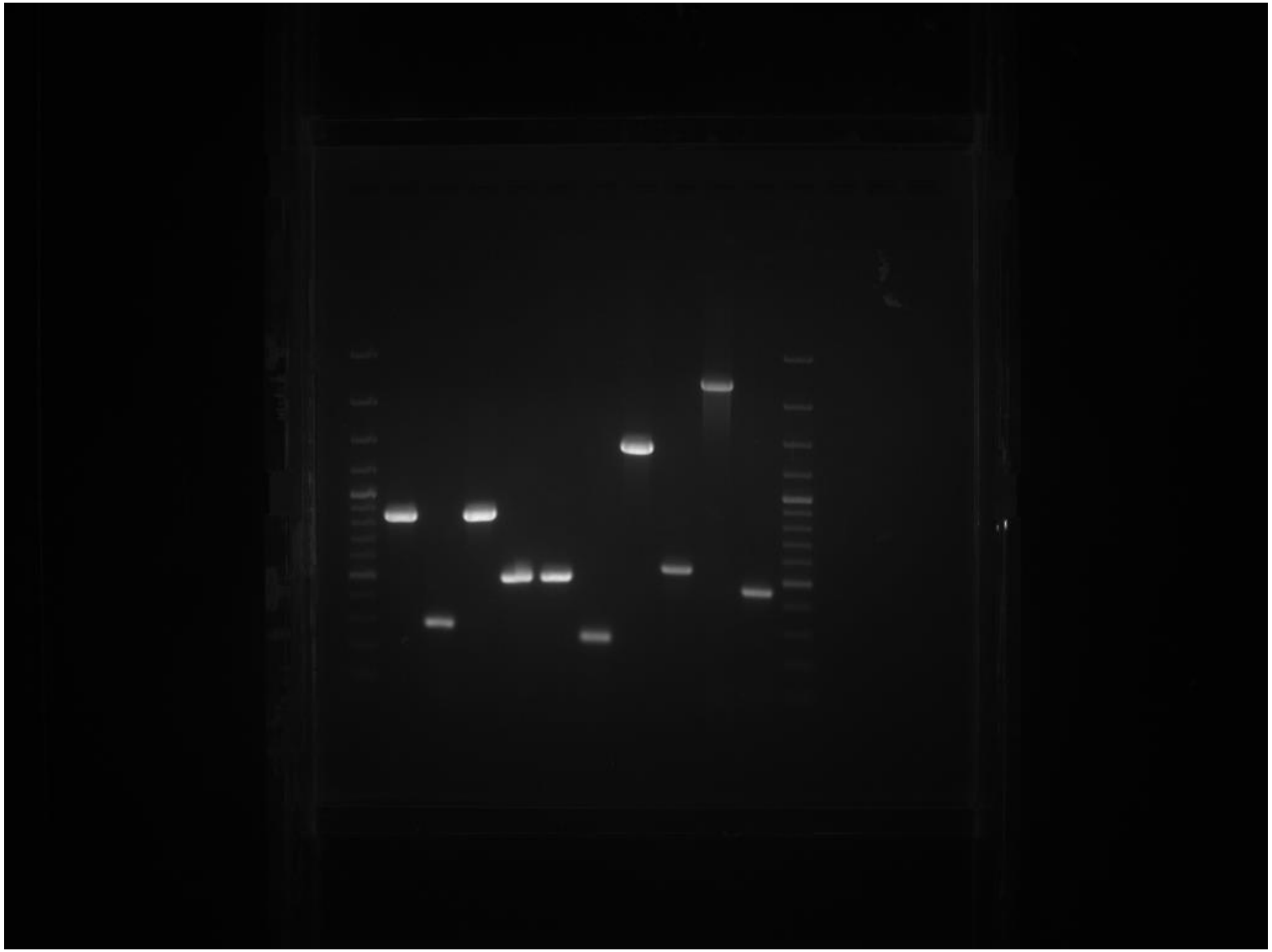

Full length gel image of Fig. 1

Supplementary Figure S2

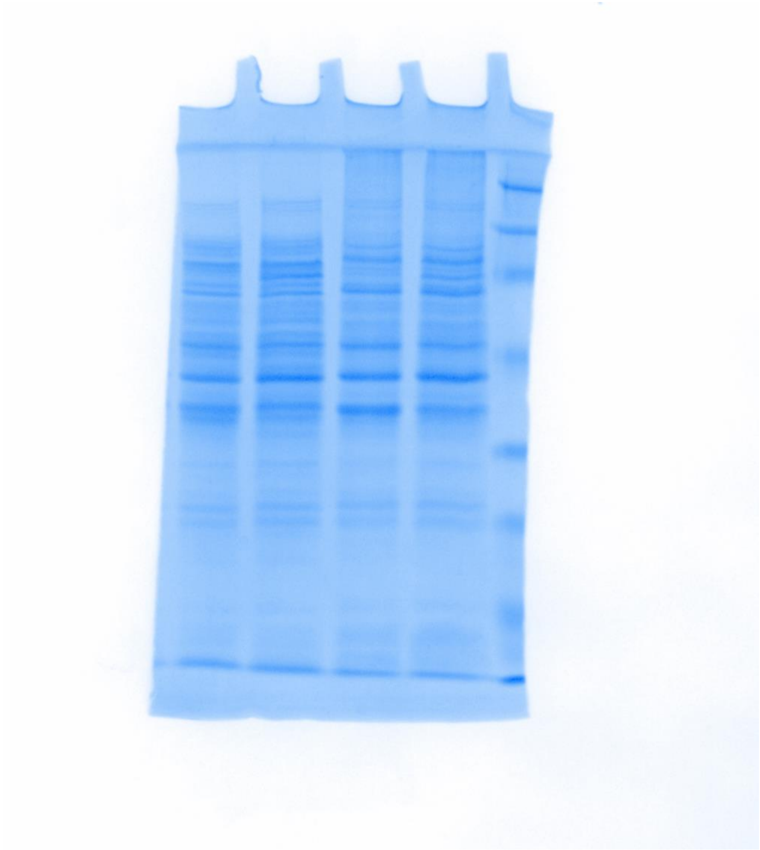

Full length gel image of Fig. 6 b.1

Supplementary Figure S3

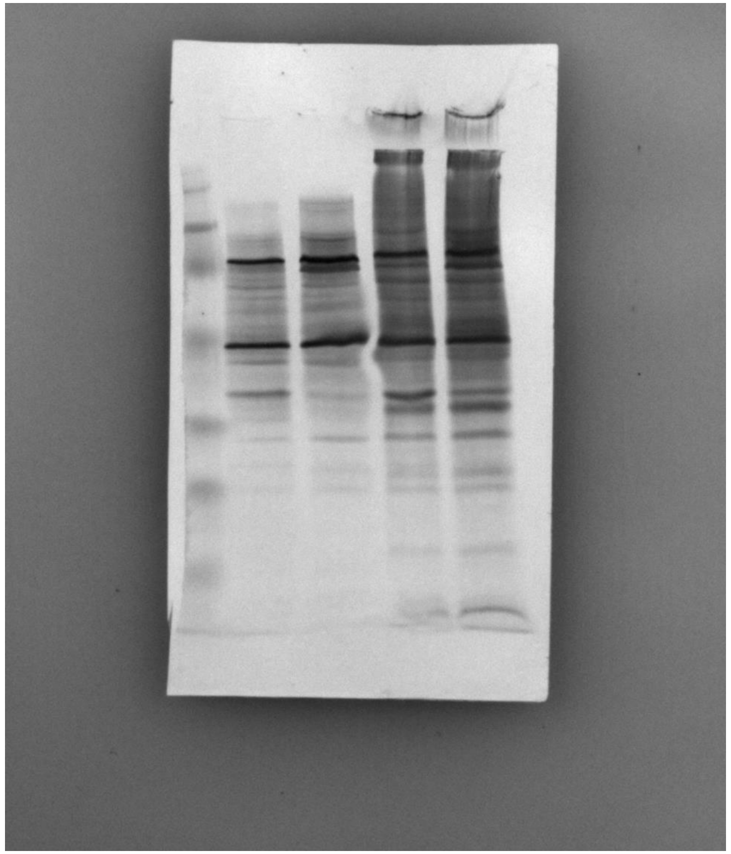

Full length gel image of Fig. 6 b.2

Supplementary Table T1

Strain	Average IntDen ± SD (n=4)	t-test
S. Typhimurium 0 mM	165.69 ± 56.77	0.0008
Δ5*msr* 0 mM	195.16 ± 59.76	
S. Typhimurium 3 mM	405.57 ± 95.99	0.001
Δ5*msr* 3 mM	465.89 ± 90.09	

ImageJ analysis of bands

Individual bands in each lane of the oxyblot were marked as regions of interest and their integrated densities were quantified using ImageJ. For each individual experiment, the sum of the integrated densities of the bands in their respective lanes was used for statistical analysis. The data is presented as mean ± SD (n=4) and analyzed by students' *t*-test.

Supplementary Figure S4

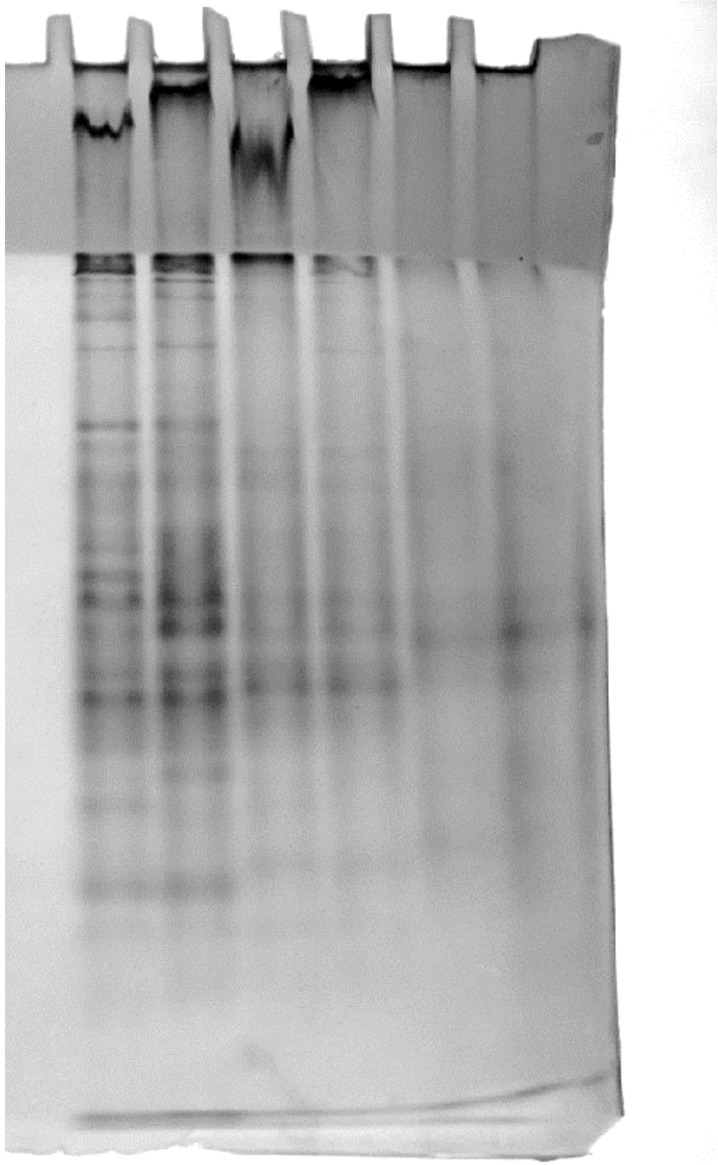

Full length gel image of Fig. 8

Supplementary Fig. S5

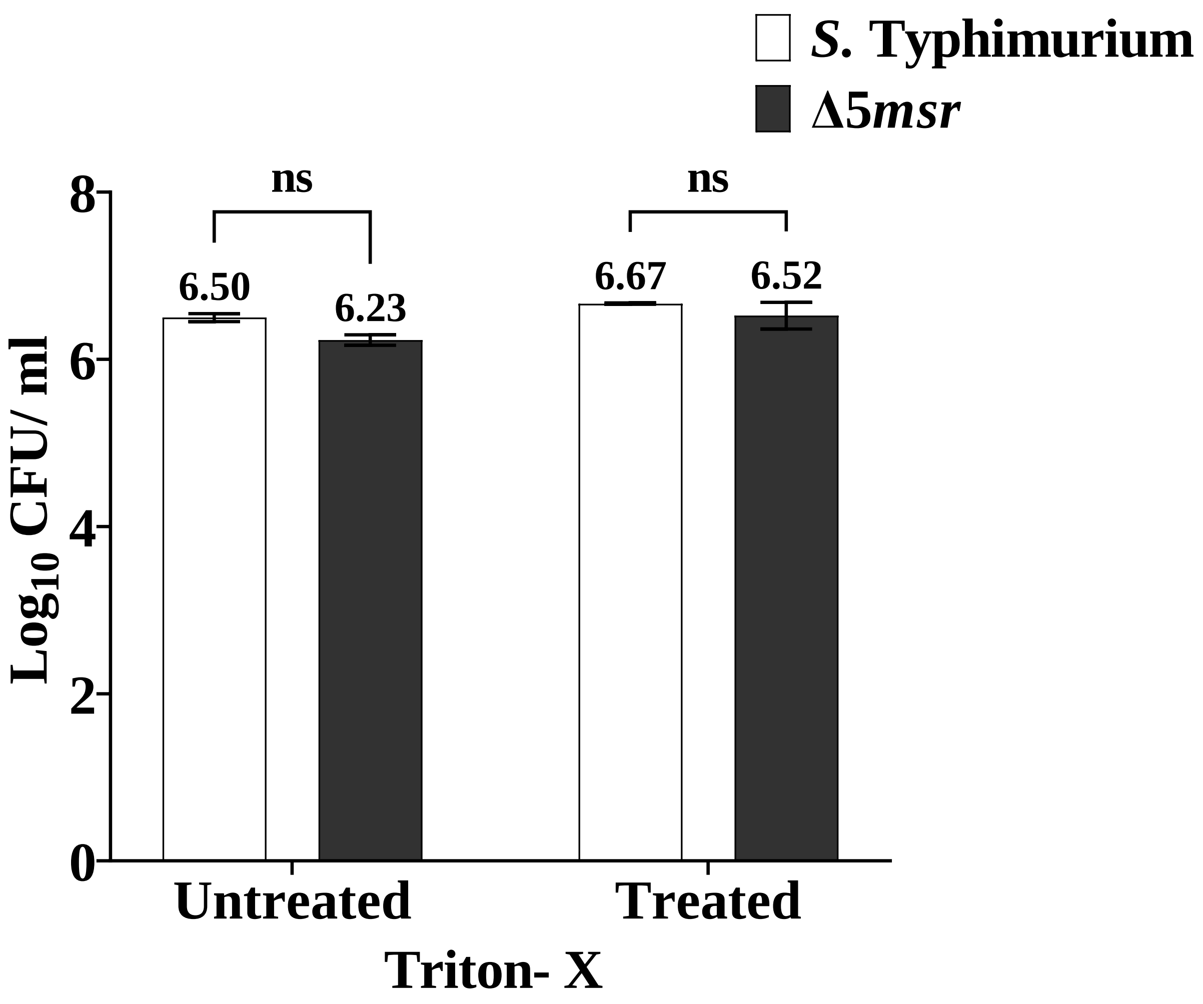

Δ5*msr* mutant or *S*. Typhimurium strains are not susceptible to Triton X-100

Δ5*msr* mutant and *S*. Typhimurium strains were incubated with 0.1 % Triton X-100. Following dilution with PBS, cultures were serially diluted and plated on agar media. CFUs were counted following incubation of the plates. The data is presented as mean ± SE (n=3) and analyzed by two-way ANOVA.
